# The *Chlamydia muridarum* Organisms Fail to Auto-Inoculate the Mouse Genital Tract after Colonization in the Gastrointestinal Tract for 70 days

**DOI:** 10.1371/journal.pone.0155880

**Published:** 2016-05-18

**Authors:** Luying Wang, Qi Zhang, Tianyuan Zhang, Yuyang Zhang, Cuiming Zhu, Xin Sun, Nu Zhang, Min Xue, Guangming Zhong

**Affiliations:** 1 The 3^rd^ Xiangya Hospital, Central South University, Changsha, Hunan 400000, P. R. China; 2 Department of Microbiology and Immunology, University of Texas Health Science Center at San Antonio, Texas, United States of America; University of Louisville, UNITED STATES

## Abstract

*Chlamydia muridarum* is known to colonize in the gastrointestinal tract for long periods of time, which has been hypothesized to serve as a reservoir for spreading to the genital tract. To test this hypothesis, a luciferase-expressing *C*. *muridarum* was used to establish a long-lasting infection in the mouse gastrointestinal tract following either intragastric or intrarectal inoculations. *In vivo* imaging revealed significant bioluminescent signals mainly in the mouse abdominal area throughout the experiments. *Ex vivo* imaging localized the signals to the mouse gastrointestinal tract, which was confirmed by monitoring the *C*. *muridarum* organisms in the mouse organs/tissues. Despite the long-lasting colonization in the gastrointestinal tract and active shedding of infectious organisms in the rectal swabs, the organisms did not cause any significant infection or pathology in the genital tract throughout the experiments, which was reproduced in multiple strains of mice and with an increased inoculation dose to the gastrointestinal tract. The above observations have demonstrated that the long-lasting *C*. *muridarum* organisms from the gastrointestinal tract are inefficient in auto-inoculating the genital tract, suggesting that the gastrointestinal tract *Chlamydia* may utilize an indirect mechanism to affect its pathogenicity in the genital tract.

## Introduction

*Chlamydia* has been detected in the gastrointestinal (GI) tracts of animals [1] and humans [2–5]. *Chlamydia muridarum* is known to last for long periods of time in the mouse GI tract [6–9]. However, the medical significance of the GI tract chlamydial infection remains unknown. Chlamydia is a sexually transmitted bacterial pathogen that causes pathologies in the genital tract [10, 11] but not associated with GI tract pathology in animals [6, 7, 12] or humans [13]. Some have proposed that the long-lasting chlamydial organisms in the GI tract may serve as a reservoir for auto-inoculating the genital tract, hence enhancing chlamydial pathogenicity in the genital tract [12]. However, this hypothesis has not been tested and there is still lack of experimental evidence for supporting this hypothesis.

*C*. *muridarum* has been extensively used for studying the mechanisms of *C*. *trachomatis* pathogenesis and immunity [14–19] because a single intravaginal inoculation of mice with *C*. *muridarum* results in hydrosalpinx and infertility [20–22], closely mimicking the tubal adhesion/hydrosalpinx/infertility observed in women [23–25]. Using this mouse model, it has been shown that adequate ascension of *C*. *muridarum* to the upper genital tract is necessary for *C*. *muridarum* induction of hydrosalpinx [22, 26–28]. However, *C*. *muridarum* infection in the mouse genital tract is self-limited while mice develop tubal fibrosis/hydrosalpinx that last long after the tubal infection is resolved [22, 26], leaving a temporal gap between the self-limited infection and the long-lasting pathology in the genital tract.

The question is whether the long-lasting *C*. *muridarum* infection in the GI tract can fill in the temporal gap by serving as a reservoir for continuously auto-inoculating the genital tract after the initial tubal infection is cleared. To address this question, in the current study, we first established a long-lasting infection with *C*. *muridarum* in the mouse GI tract and confirmed that the GI tract *C*. *muridarum* was restricted to the GI tract by using *in vivo*/*ex vivo* imaging and chlamydial organism detection in the organs/tissues. These mice continuously shed infectious *C*. *muridarum* organisms from the GI tracts. We then monitored the genital tract infection and inflammation in the same mice. No significant and consistent live chlamydial organisms were detected in the vaginal swabs of these mice. More importantly, none of the mice developed any significant hydrosalpinx when examined macroscopically or inflammatory infiltration in the genital tract tissue when examined microscopically. These observations, reproduced both in multiple strains of mice and when an increased inoculation dose was applied to the gastrointestinal tract, have led us to conclude that the long-lasting *C*. *muridarum* organisms in the GI tract cannot effectively auto-inoculate the genital tract or cause pathology in the upper genital tract. Thus, alternative mechanisms should be sought for addressing the potential contributions of the GI tract chlamydial infection to the chlamydial pathogenicity in the genital tract.

## Materials and Methods

### Chlamydial organism growth

*C*. *muridarum* (CM) strain Nigg organisms (initially acquired from Dr. Robert C Brunham’s lab at the University of Manitoba in 1999) were propagated and purified in HeLa cells (human cervical carcinoma epithelial cells, ATCC cat# CCL2) as described previously [29, 30]. The full genome sequence of this *C*. *muridarum* strain designated as Nigg3 is available under Genbank accession# CP009760.1. A passaged clone designated as Nigg3G13.32.1 was isolated from the Nigg3 stock [31]. The Nigg3 stock was also used to cure of plasmid and a resultant plasmid-free clone CMUT3G5 (for full genome sequence: Genbank accession# CP006974.1) was used for transformation with pGFP-luci-CM plasmid [32] as described previously [33, 34]. The genome sequences of the *C*. *muridarum* Nigg3G13.32.1 and the luciferase-expressing CMUT3G5-pGFP-Luci are nearly identical with the only exception in the gene coding for TC0412. Although variations in this gene from *C*. *trachomatis* were found to affect the infectivity of *C*. *trachomatis* in the mouse genital tract [35], mutations in this gene were shown to have no significant effect on either the infectivity or the pathogenicity of *C*. *muridarum* in the mouse genital tract [31, 36]. Both Nigg3G13.32.1 and CMUT3G5-pGFP-Luci organisms were propagated in HeLa cells and purified as elementary bodies (EBs) as mentioned above. Aliquots of the purified EBs were stored at -80oC until use.

### Mouse infection and *in vivo* and *ex vivo* imaging

The animal experiments were carried out in accordance with the recommendations in the Guide for the Care and Use of Laboratory Animals of the National Institutes of Health. The protocol was approved by the Committee on the Ethics of Laboratory Animal Experiments of the University of Texas Health Science Center at San Antonio.

Purified *C*. *muridarum* EBs were used to infect six to seven week-old female mice (Jackson Laboratories, Inc., Bar Harbor, Maine) intragastrically (i.g.), intrarectally (i.r.) or intravaginally (i.vag.) with 5 X 10^4^ or 1 X 10^7^ inclusion-forming units (IFUs) as described previously [22, 30] and indicated in individual experiments. The following three mouse strains were used in the current study: CBA/J (Jackson Laboratories stock number 000656), C57BL/6J (000664) and C3H/HeJ (000659). Five days prior to infection, each mouse was injected with 2.5 mg medroxyprogesterone (Depo-Provera; Pharmacia Upjohn, Kalamazoo, MI) subcutaneously. After infection, mice were subjected to *in vivo* imaging, monitored for vaginal and rectal live organism shedding, and sacrificed on different days post-infection for *ex vivo* imaging and quantitating *C*. *muridarum* live organisms or genome copies in different organs or tissues.

Mice infected with the luciferase-expressing *C*. *muridarum* clone CMUT3G5-pGFP-Luci were imaged using the Xenogen IVIS imaging system (PerkinElmer, Hopkinton, MA) on different days after infection. Prior to imaging, 500μl of D-luciferin (40mg/ml in sterile PBS) were intraperitoneally injected into each mouse. Twenty to 30 minutes after injection, mice were anesthetized with 2% isofluorane. Bioluminescence images of the whole mouse bodies were captured as described previously [30, 32]. Immediately after the whole body imaging, mice were sacrificed by using an overdose of isofluorane followed by cervical dislocation. The mouse organs were taken out and placed in a Petri dish for *ex vivo* imaging. Intensity of bioluminescence was analyzed by using a Living Image software from PerkinElmer.

### Titrating live chlamydial organisms recovered from swabs and tissue homogenates

For monitoring live organism shedding, vaginal and rectal swabs were taken every three to four days for the first week and weekly thereafter. To quantitate live chlamydial organisms, each swab was soaked in 0.5 mL of SPG, vortexed with glass beads, and the chlamydial organisms released into the supernatants were titrated on HeLa cell monolayers in duplicate. The infected cultures were processed for immunofluorescence assay as described below. Inclusions were counted in five random fields per coverslip under a fluorescence microscope. For coverslips with less than one IFU per field, entire coverslips were counted. Coverslips showing obvious cytotoxicity of HeLa cells were excluded. The total number of IFUs per swab was calculated based on the mean IFUs per view, the ratio of the view area to that of the well, dilution factor, and inoculation volumes. Where possible, a mean IFU/swab was derived from the serially diluted and duplicate samples for any given swab. The total number of IFUs/swab was converted into log_10_ and used to calculate the mean and standard deviation across mice of the same group at each time point.

For quantitating live organisms from mouse organs and tissue segments, immediately after *ex vivo* imaging, each organ or tissue segment was transferred to a tube containing 0.5 to 5ml SPG depending the sizes of the organs. Each genital tract was cut into 5 segments/portions including vagina/cervix (CV), left uterine horn (L-uh), right uterine horn (R-uh), left oviduct/ovary (L-ov) and right oviduct/ovary (R-ov). Each GI tract was divided into 5 segments including stomach, small intestine (SI), ceccum, colon and ano-rectum (rectum). The organs and tissue segments were homogenized in cold SPG using a 2mL tissue grinder (cat# K885300-0002, Fisher scientific, Pittsburg, PA) or an automatic homogenizer [Omni Tissue Homogenizer, TH115, Kennesaw, GA]. The homogenates were further briefly sonicated and spun at 3000 rpm for 5min to pellet remaining large debris. The supernatants were titrated for live *C*. *muridarum* organisms on HeLa cells as described above. The results were expressed as log_10_ IFUs per organ or tissue segment.

The immunofluorescence assay used for titrating live organisms were carried out as described previously [27, 28]. For titrating the live organisms recovered from a given sample, the mean number of inclusions per view was derived from counting five random views. The total number of live organisms in a given sample was calculated based on the mean inclusions per view, ratio of view area to that of the well, dilution factor, and inoculum volume and expressed as log_10_ IFUs per sample.

### Titrating the number of *C*. *muridarum* genomes in the mouse samples using quantitative PCR

To quantitate the genome copies of *C*. *muridarum*, a portion of each tissue homogenate was transferred to the lysis buffer provided with Quick-gDNA miniPrep Kit (Cat#: 11-317C, Genesee Scientific, San Diego, CA) and subjected to DNA extraction following manufacturer’s instruction. Each DNA preparation was eluted in 100μl elution buffer, and 2μl was used for quantitative PCR (qPCR). The following primers derived from the Chlamydia 16S rRNA coding region were used: forward primer (5′-CGCCTGAGGAGTACACTCGC-3), reverse primer, (5′-CCAACACCTCACGGCACGAG-3‘) and Double-Quenched Probe (5′-CACAAGCAGTGGAGCATGTGGTTTAA-3′) (Integrated DNA Technologies, Coralville, Iowa). PCR was carried out in a total volume of 10 μ in a CFX96 Touch Deep Well Real-Time PCR Detection System with iQ Supermix real-time PCR reagent (Bio-Rad, Hercules, CA). Genome copy numbers for a given sample in triplicate were calculated based on a standard plasmid DNA prep in the corresponding samples. The qPCR conditions included an initial denaturation step at 95°C for 3 minutes, followed by 40 cycles of amplification at 95°C for 15 seconds and 60°C for 1 minute.

### Evaluating genital tract gross pathology and inflammatory infiltration

Seventy days after infection, mice were euthanized for harvesting genital tracts. Gross pathology of hydrosalpinx was observed and documented by high-resolution digital photography. Hydrosalpinx was scored according to an ordinal scale where 0 indicates no hydrosalpinx, 1 indicates hydrosalpinx that is only observable under magnification, 2 indicates visible hydrosalpinx smaller than the size of the oviduct, 3 indicates hydrosalpinx roughly equal to the size of the oviduct, and 4 indicates hydrosalpinx larger than the oviduct. Bilateral hydrosalpinx severity was calculated for each mouse as the summed scores of the left and right oviducts. Hydrosalpinx incidence was calculated as the number of mice with a bilateral score of 1 or higher divided by the total number of mice in the group. Chronic inflammation or infiltration of mononuclear cells into the genital tract tissues was evaluated histologically. Following gross pathology assessment, whole genital tracts were fixed in 10% neutral formalin, stored in 70% ethanol in water, embedded in paraffin, and serially sectioned longitudinally across the plane of the whole genital tract at 5-μm intervals on an AccuCut SRM 200 microtome (Sakura, Torrance, CA). Three nearly equidistant sections at roughly 5 μm, 30 μm, and 55 μm into the lateral surface of the oviduct were subjected to hematoxylin and eosin (H&E) staining, sealed on glass tissue slides, and observed under a microscope for infiltration of mononuclear inflammatory cells into tissue surrounding the oviduct. These infiltrates were scored on an ordinal scale where 0 represents no cellular foci, 1 indicates a single focus, 2 indicates two to four loci, 3 indicates more than four foci, and 4 indicates confluent infiltration. The median of the three scores served as a single value for each oviduct unilateral inflammation score, and both unilateral scores for each mouse were combined to form a bilateral score. Bilateral oviduct dilation scores were acquired from the same three sections of each oviduct in the same manner as the inflammation scores but using the criteria for scoring hydrosalpinx described above.

### Statistics analyses

The incidence rates were analyzed using Fisher’s exact test while the number of live organisms (IFUs), genome copies and pathology scores were analyzed using the Wilcoxon rank sum test for comparing any two groups and Kruskal-Wallis for comparing more than two groups.

## Results

### Both intragastric and intrarectal inoculations with *C*. *muridarum* lead to a long lasting infection in the mouse GI tract

**Fig 1:**
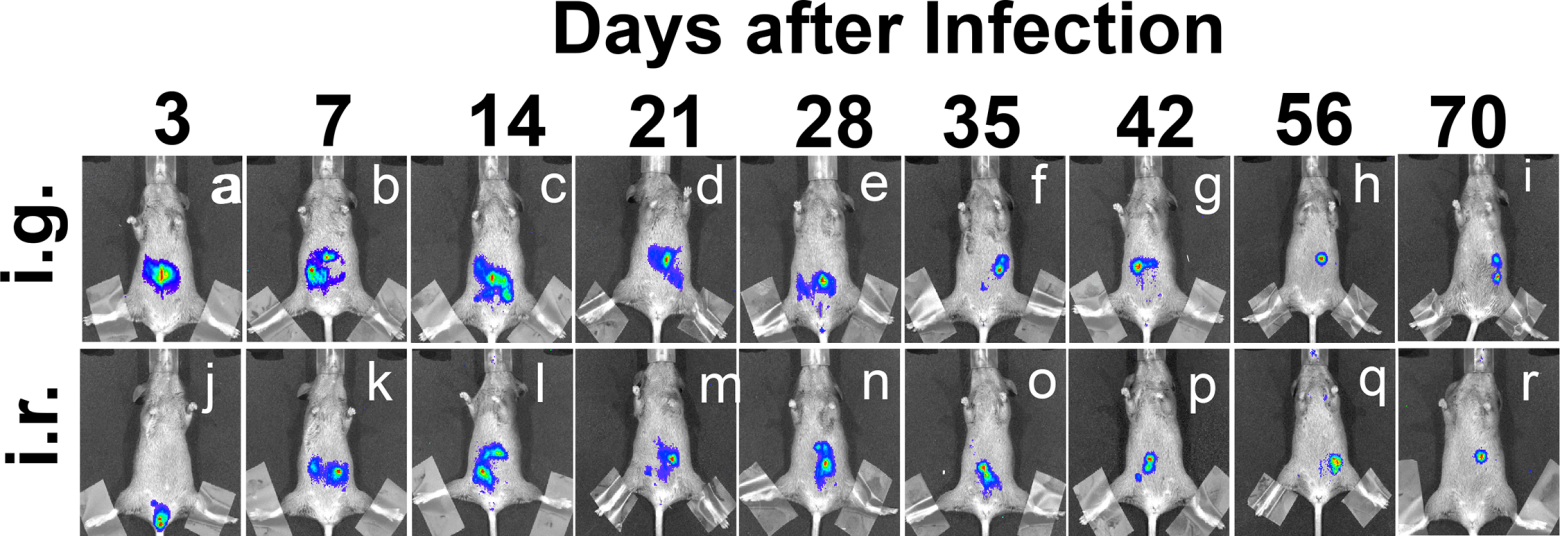
*In vivo* imaging of mice infected intragastrically or intrarectally with luciferase-expressing *C*. *muridarum*. CBA/J mice were intragastrically (i.g., n = 5, panels a-i) or intrarectally (i.r., n = 5, j-r) inoculated with 5 x 10^4^ IFUs of luciferase-expressing *C*. *muridarum*. On different days after the inoculations as indicated on top of the figure, a whole body *in vivo* imaging was used to detect the luciferase-generated bioluminescence signals as displayed in red/green/blue colors (in the order of decreasing intensity). Images taken from one of the mice in each group were shown. It is worth noting that the bioluminescence signals were detectable as early as day 3 after inoculation at the initial inoculation sites either stomach (panel a) or anorectum (j). Starting on day 7, the signals persisted in the mouse abdominal area throughout the experiments with similar distribution patterns regardless of the initial inoculation routes.

**Fig 2:**
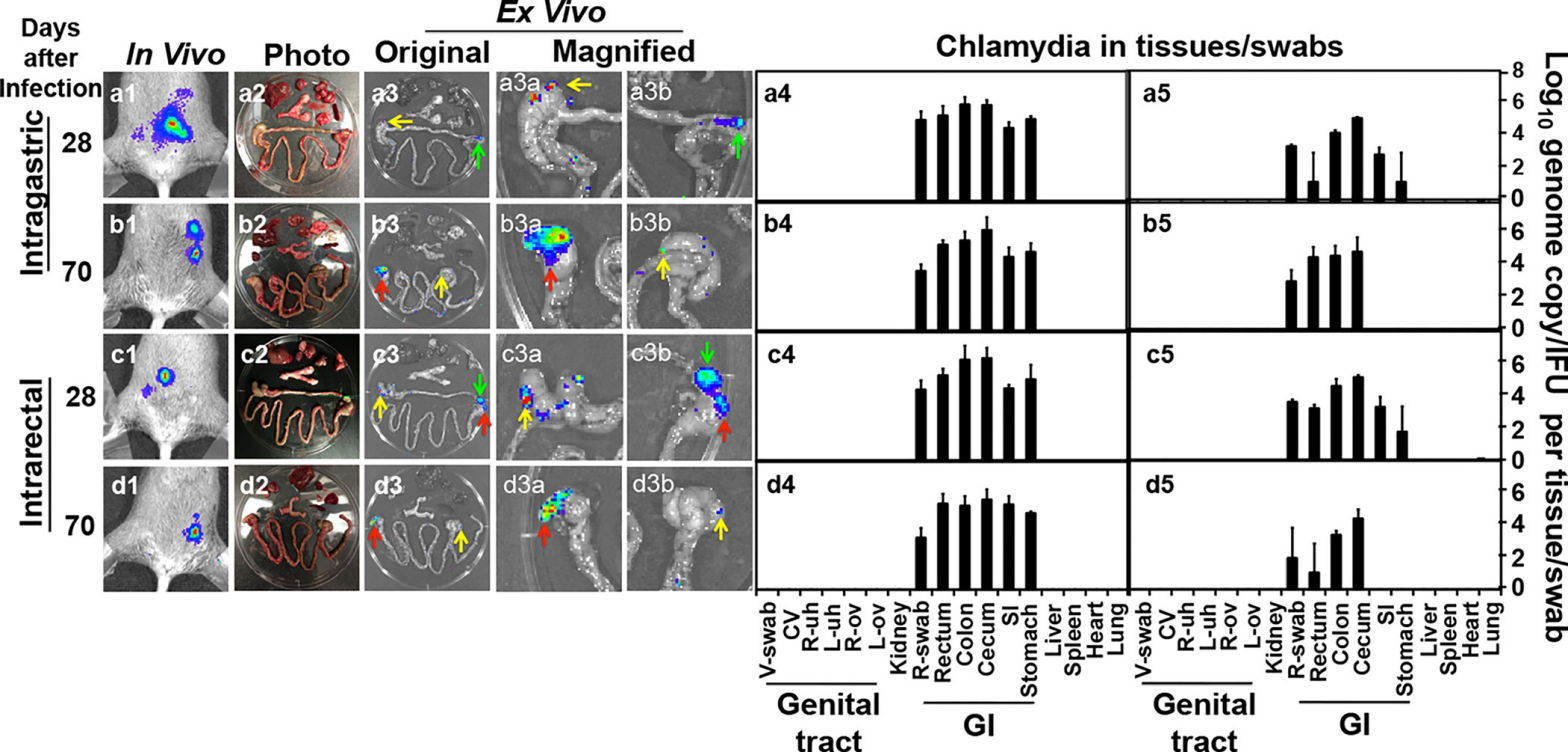
*Ex vivo* imaging of luciferase-expressing *C*. *muridarum* and recovery of *C*. *muridarum* genomes and infectious organisms from mouse organs. CBA/J mice were intragastrically (panels a1-5 & b1-5) or intrarectally (panels c1-5 & d1-5) inoculated with 5 x 10^4^ IFUs of luciferase-expressing *C*. *muridarum* as described in Fig 1 legend. On days 28 & 70 after infection as indicated on the left of the figure, a whole body *in vivo* imaging was taken (panels a1-d1). At the same time, a group of 3 to 5 mice were sacrificed at each time point for harvesting the internal organs. Organs from the same mouse were arrayed in a Petri dish as shown in panels a2-d2. *Ex vivo* imaging was immediately taken to acquire bioluminescence signals as shown in panels a3-d3 (column labeled with “original”). Green arrows point to signals from the rectum, yellow to the ceccum and red to the stomach. The arrow-pointed areas were magnified and displayed on the right with the same arrow indication (columns labeled with “magnified”, panels a3a-d3b). At each time point, only one representative mouse-derived *in vivo* whole body image and *ex vivo* image of the organs from the same mouse are shown. The same organs after *ex vivo* imaging were subjected to tissue homogenization with some organs separated into different tissues as shown along the X-axis at the bottom. On the same day of sacrificing, both vaginal and rectal swabs were taken from each mouse. All organs/tissues/swabs, including lungs, heart, spleen, liver, stomach, small intestine (SI), ceccum, colon, rectum, rectal (R)-swab (from stomach to R-swab, grouped as gastrointestinal tract, or GI), kidneys, left (L) or right (R) ovary-oviduct (ov) or uterine horn (uh), vagina-cervix (CV) and vaginal (V) swab (from L-ov, grouped as genital tract) were titrated for *Chlamydia muridarum* genome copies (panels a4-d4) and live organisms (as inclusion forming units, IFUs, panels a5-d5). The genome copies and IFUs were expressed in total numbers per organ/tissue/swab (log10) as mean ± standard error and displayed along the Y-axis on the right. Note that bioluminescence signals were mainly detected in the mouse gastrointestinal tract throughout the experiments regardless of the inoculation routes, which is confirmed by the detection of *C*. *muridarum* genomes and live organisms from the same organs/tissues. The *C*. *muridarum* organisms detected from the rectal and vaginal swabs reflect the chlamydial presence in the GI and genital tract respectively.

We used a whole body *in vivo* imaging technology for monitoring the distribution of the luciferase-expressing *C*. *muridarum* organisms in CBA/J mice following an intragastric or intrarectal inoculation (Fig 1). The luciferase-generated bioluminescence signal was detected as early as day 3 after the inoculation at the sites of inoculation. By day 7, the signal started to spread around the stomach area following an intragastric inoculation while the signal ascended to the abdominal area following an intrarectal inoculation. Regardless of the inoculation routes, the signals persisted in the abdominal area throughout the experiments. To test whether the luciferase-expressing *C*. *muridarum* organisms were restricted to the gastrointestinal tract only or also spread to other organs/tissues, we monitor the organism distribution by both using an *ex vivo* imaging of the mouse organs and directly detecting the *C*. *muridarum* organisms in the corresponding organs/tissues on days 28 and 70 after the initial inoculation (Fig 2). When all the organs/tissues including lung, heart, spleen, liver, the GI tract, kidney and the genital tract were subjected to the *ex vivo* imaging, we found that the bioluminescence signals were only from the GI tract tissues, such as the rectum, cecum and stomach. Homogenates made from the same organs/tissues, together with the rectal and vaginal swabs, were titrated for both *C*. *muridarum* genomes using qPCR and live organisms using an immunofluorescence assay. We found that significant levels of *C*. *muridarum* genomes and live organisms were only detected from the GI tissues/swabs. Together, the above observations have demonstrated that the long-lasting persistence of *C*. *muridarum* organisms is restricted to the GI tract only.

### The long-lasting *C*. *muridarum* infection in the GI tract fails to cause any significant infection or pathology in the mouse genital tract

**Fig 3:**
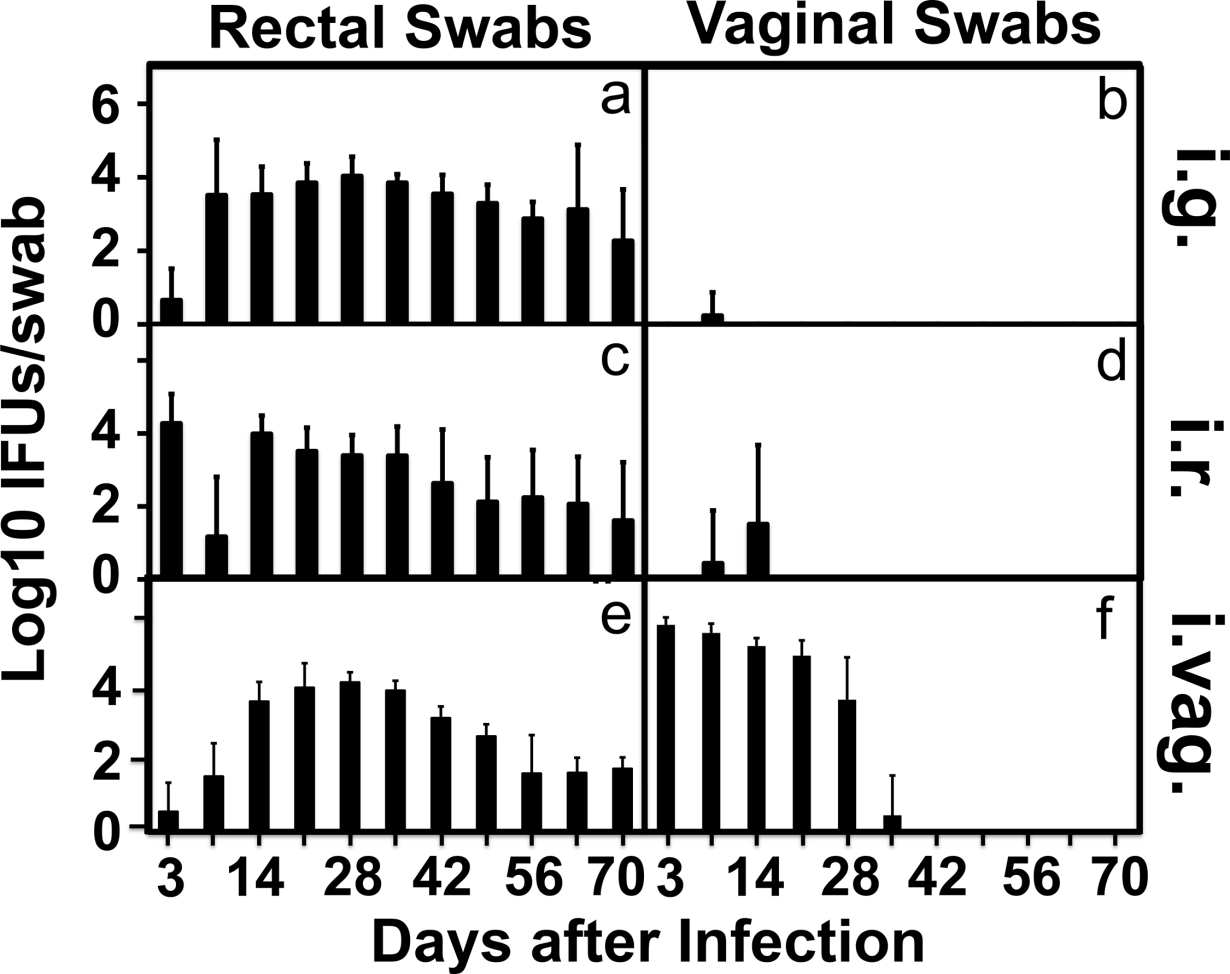
Recovering live *C*. *muridarum* organisms from GI versus genital tracts of CBA/J mice. The same two groups of mice as described in Fig 1 legend and an additional group of CBA/J (n = 5) infected intravaginally (i.vag.) with the same amount of luciferase-expressing *C*. *muridarum* were monitored for live chlamydial organism shedding in their rectal (panels a, c & e) and vaginal (b, d & f) swabs on different days after infection as indicated along the X-axis at the bottom of the figure. The results were expressed as Log10 IFUs displayed along the Y-axis. Note that no significant live organisms were detected in the vaginal swabs of mice infected in the GI tract throughout the experiments.

**Fig 4:**
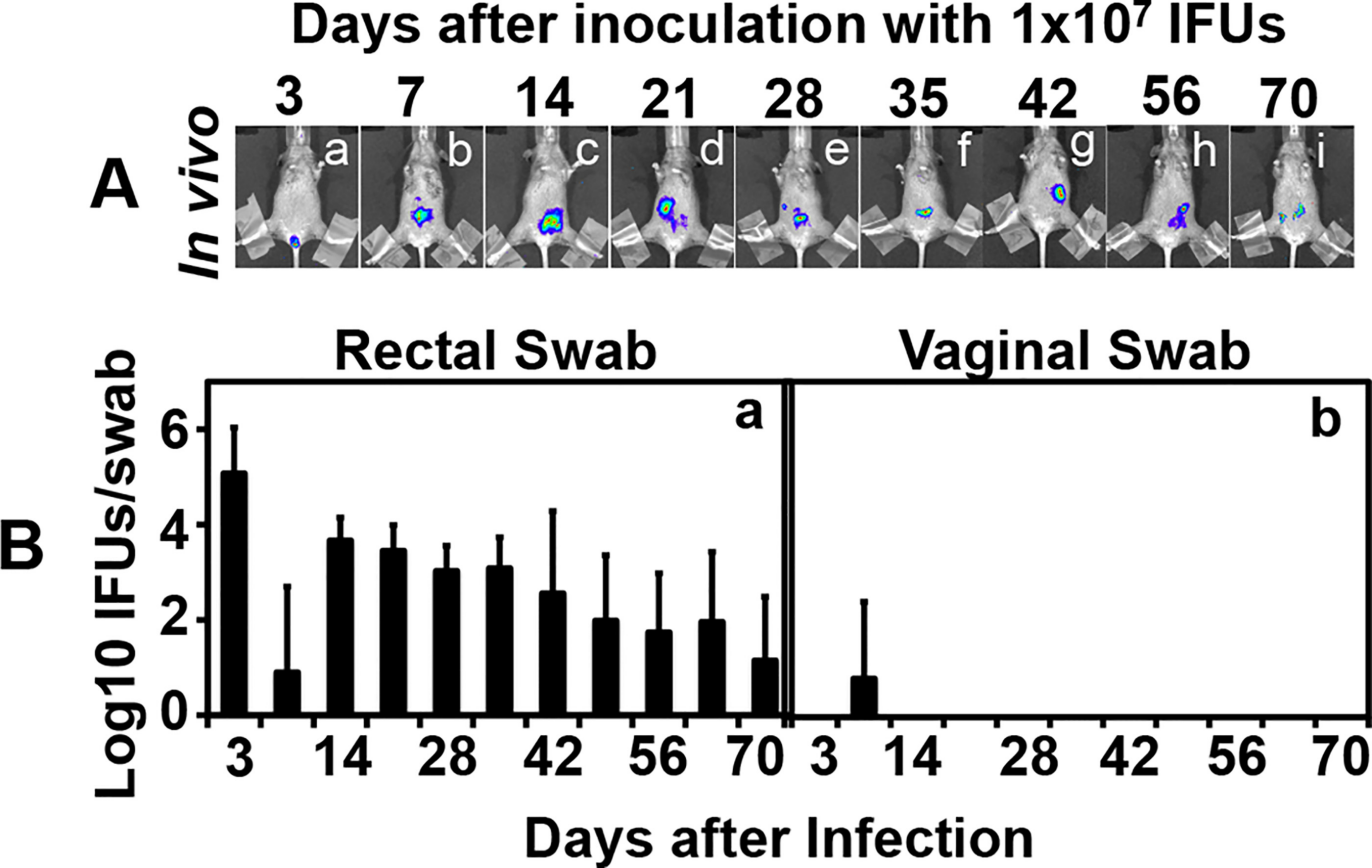
Monitoring *C*. *muridarum* infection in both the GI and genital tracts following an intrarectal inoculation with 1 x 10^7^ IFUs. CBA/J mice were intrarectally (n = 4) inoculated with 1 x 10^7^ IFUs of luciferase-expressing *C*. *muridarum*. (A) On different days after the inoculations as indicated on top of the figure, a whole body *in vivo* imaging was used to detect the luciferase-generated bioluminescence signals as displayed in red/green/blue colors (in the order of decreasing intensity). Images taken from one of the mice in each group were shown. (B) At the same time, these mice were monitored for live chlamydial organism shedding in their rectal (panel a) and vaginal (b) swabs on different days after infection as indicated along the X-axis at the bottom of the figure. The results were expressed as Log10 IFUs displayed along the Y-axis. Note that no significant live organisms were detected in the vaginal swabs throughout the experiment.

**Fig 5:**
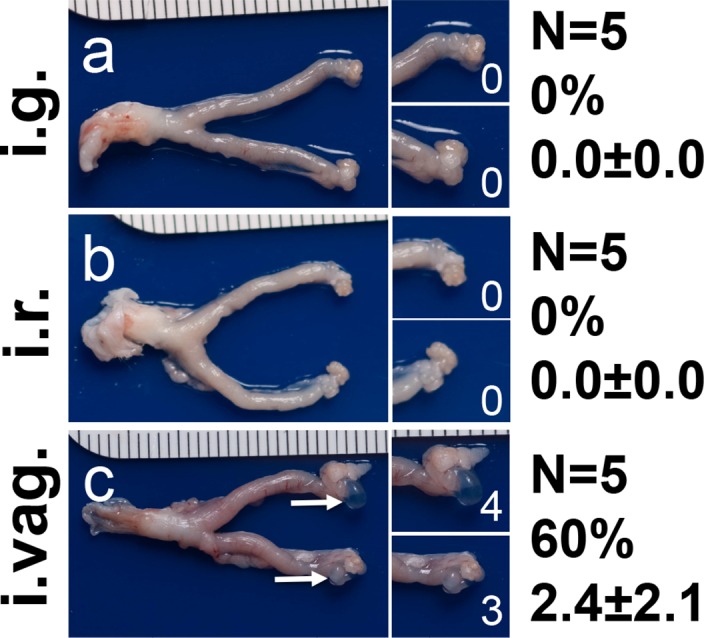
Evaluating the gross pathology in the genital tracts of CBA/j mice infected with *C*. *muridarum*. The three parallel groups of CBA/J mice infected with luciferase-expressing *C*. *muridarum* intragastrically (i.g., panel a, n = 5), intrarectally (i.r., b, n = 5) or intravaginally (i.vag., panels c, n = 5) as described in Fig 3 legend were sacrificed on day 70 after infection for visually identifying (arrow) and scoring (white numbers) hydrosalpinx. Mice with hydrosalpinx on either side of the genital tract were defined positive for hydrosalpinx. The severity of hydrosalpinx was scored from each oviduct independently and the scores from both sides of the same mouse were added together as the score for that mouse. Both incidence rates and severity scores of hydrosalpinx from each group were listed beside the corresponding images. Note that significant hydrosalpinx was detected only in mice infected intravaginally but intragastrically or intrarectally.

**Fig 6:**
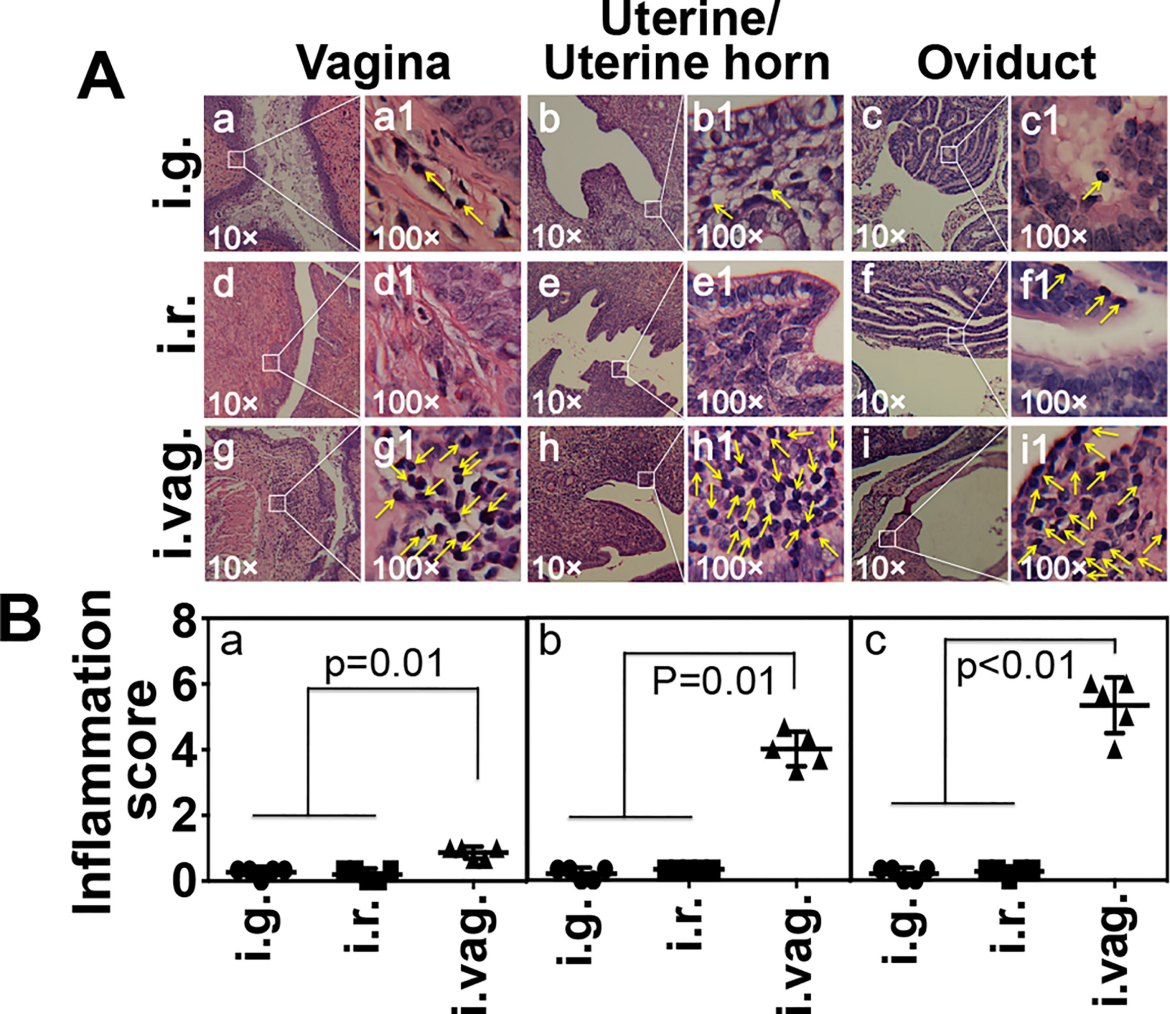
Evaluating the microscopic pathology in the mouse genital tracts. The same mouse genital tissues harvested on day 70 after infection as described in Fig 5 legend were examined for inflammatory infiltration in the genital tract tissues. (A) Representative images of H&E-stained sections of vagina (panels a, d & g under 10x objective lens while a1, d1 & g1 under 100x), uterine/uterine horn (b, e & h, 10x; b1, e1 & h1, 100x) and oviduct (c, f & i, 10x; c1, f1 & i1, 100x) from mice infected intragastrically (i.g., a-c), intrarectally (i.r., d-f) and intravaginally (i.vag., g-i) were shown. (B) The Inflammatory infiltration from the vagina (a), uterine/uterine horn (b) and oviduct (c) tissues were semi-quantitatively scored (shown along the Y-axis) as described in the Materials and Method section. Kruskal-Wallis Test was used to calculate the p values listed in the figure. Note that only the tissues from the intravaginally infected mice displayed significant levels of inflammatory infiltration.

**Fig 7:**
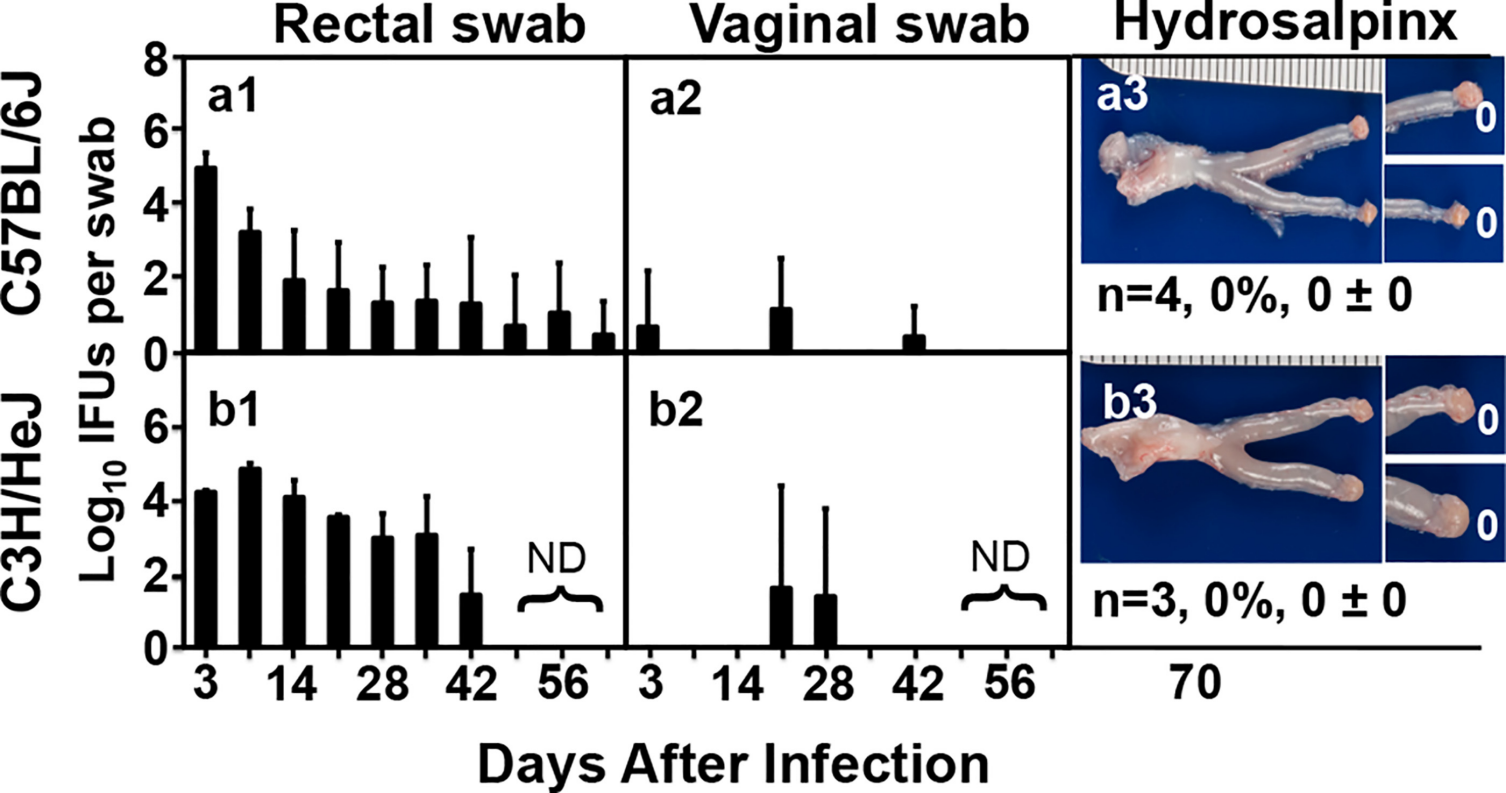
Monitoring *C*. *muridarum* infection in the GI and genital tracts of C57BL/6J and C3H/HeJ mice following an intrarectal inoculation. C57BL/6J (panels a1-a3, n = 4) and C3H/HeJ (b1-3, n = 3) mice were intrarectally infected with *C*. *muridarum* and both anorectal and cervico-vaginal swabs were taken on different days after infection as listed along the X-axis in the bottom of the figure. The number of live organisms recovered from the swabs was expressed as Log10 IFUs. "ND” denotes not detected. On day 70 after infection, mice were sacrificed for harvesting the genital tracts for visually identifying and scoring hydrosalpinx (a3 & b3) as described in Fig 3 legend. Note that although significant levels of live organisms were continuously detectable in the mouse GI tract throughout the experiment, no significant level of live organisms was detected in the mouse vaginal swabs and there was no significant hydrosalpinx in any of the mice.

To address whether the long-lasting *C*. *muridarum* infection in the GI tract can serve as a reservoir for auto-inoculating the genital tract, we carefully monitored both the GI tract and genital tract chlamydial organism shedding over time from the CBA/J mice inoculated with *C*. *muridarum* via intragastric, intrarectal or intravaginal routes (Fig 3). The intragastrically or intrarectally inoculated mice developed significant levels of live organism shedding in the rectal swabs with a peak level on day 3 for the intrarectally inoculated mice and after day 7 for the intragastrically inoculated mice. The live shedding lasted throughout the experiments in both groups of mice. However, no significant live organisms were detected in the vaginal swabs of these mice, indicating that the gastrointestinal *C*. *muridarum* organisms did not effectively auto-inoculate the genital tracts. The minimal levels of live organisms detected in one or two vaginal swabs harvested on day 7 or 14 may be caused by swabbing contamination since the chlamydial organisms were not detected in any two consecutive swabs from the same mice. On the contrary, the intravaginally inoculated mice displayed robust live organism recoveries in both the rectal and vaginal swabs and the live organisms continued to shed in the rectal swabs even after the vaginal swabs were cleared, which is consistent with what we have previously reported [30]. It is clear that although the *C*. *muridarum* organisms can readily spread from the genital tract to the GI tract [30], these organisms cannot effectively auto-inoculate the genital tract from the GI tract. This conclusion is still true even when we increased the GI tract inoculation by 200 folds (Fig 4). We found no significant genital tract infection in mice intrarectally inoculated with 1 X 10^7^ IFUs of *C*. *muridarum*. We further compared both the macroscopical (Fig 5) and microscopical (Fig 6) pathologies in the genital tracts of the mice described in Fig 3. Although the intravaginally inoculated mice developed significant levels of hydrosalpinx, none of the mice inoculated with *C*. *muridarum* into the GI tract developed any hydrosalpinx. At the microscopic level, no significant inflammatory infiltration was observed in the genital tissues of the mice with the GI tract-inoculation although significant inflammatory infiltration was detected in vagina, uterine and oviduct tissues of the intravaginally infected mice. To test whether the lack of autoinoculation detected in the CBA/J mice is only restricted to this strain of mice or a universal phenomenon, we repeated the experiments in both C57BL/6J and C3H/HeJ mice (Fig 7). Both strains were inoculated with the G13.32.1 *C*. *muridarum* organisms via an intrarectal route and monitored for the presence of live organisms in the rectal and vaginal swabs. Significant levels of live *C*. *muridarum* organisms were only detected in the rectal but not vaginal swabs. Again, the scattered sheddings detected in the vaginal swabs might be caused by swabbing contamination. More importantly, no hydrosalpinx was detected in any of these mice. Thus, the lack of autoinoculation from the GI tract to the genital tract was reproduced in multiple strains of mice.

## Discussion

The *C*. *muridarum* organisms can last for long periods of time in the mouse GI tract [6–9]. In the current study, we have presented experimental evidence for addressing two questions: whether *C*. *muridarum* can persist for long periods of time only in the GI tract and whether the long-lasting *C*. *muridarum* organisms in the GI tract can effectively auto-inoculate the genital tract and contribute to the *C*. *muridarum* pathogenicity in the upper genital tract. We have shown that after the luciferase-expressing *C*. *muridarum* organisms were introduced into the GI tract via either intragastric or intrarectal inoculation, the organisms only colonized the GI tract for long periods of time. First, the whole body *in vivo* imaging revealed that the luciferase-generated signals were restricted to the abdominal area throughout the experiments; Second, the *ex vivo* imaging of all organs only detected the bioluminescent signals in the GI tract on days 28 and 70 after the inoculation; Finally, the *C*. *muridarum* organisms were detected only in the GI tract but not other organ tissues on days 28 and 70, confirming the specificity of the luciferase-generated bioluminescent signals. These observations together have both confirmed previous observations that *C*. *muridarum* infection in the GI tract can last for long periods of time [6–9] and demonstrated that the GI tract inoculation with *C*. *muridarum* via either intragastric or intrarectal routes can only lead to a long-lasting infection in the GI tract but not other organs.

Chlamydia has been detected in the GI tracts of animals [1] and humans [2–5]. However, the medical significance of the chlamydial infection in the GI tract remains unclear. Some have proposed that the GI tract Chlamydia may serve as a reservoir for auto-inoculating into the genital tract to promote chlamydial pathogenicity in the upper genital tract [9, 12]. To test the hypothesis, we first established a long-lasting infection with *C*. *muridarum* in the mouse GI tract via either intragastric or intrarectal inoculation and confirmed that the long-lasting *C*. *muridarum* in the GI tract is only restricted to the GI tract. We then monitored the genital tract infection and inflammation in mice that continuously shed live infectious organisms from their rectal swabs. We found no significant levels of *C*. *muridarum* organisms from the vaginal swabs of the same mice. More importantly, no significant pathology and inflammation were detected in the genital tract of these mice. These observations were reproduced in different strains of mice infected with the *C*. *muridarum* G13.32.1 clone. It is clear that active shedding of live *C*. *muridarum* organisms from the GI tract not necessarily auto-inoculate the genital tract in mice. Although minimal levels of live organisms were detected in the some vaginal swabs, these incidental positive detections might be caused by the swabbing contamination because the positive detection was rarely found in two or more consecutive swabs from the same mouse. More importantly, these mice did not show any sign of inflammation in the genital tract tissues.

Besides reproducing the lack of autoinoculation observation in multiple strains of mice, we also tested whether an increased inoculation dose to the mouse GI tract could facilitate autoinoculation. However, when 1 x 10^7^ IFUs of *C*. *muridarum* were delivered to mouse GI tracts, the levels of live organism shedding remained the same as those from mice intrarectally inoculated with 5 x 10^4^ IFUs, suggesting that an inoculation dose of 5 x 10^4^ IFUs was already at the saturation level and was sufficient for achieving a maximal level of infection in the mouse GI tract. It is not clear why the mouse GI tract was saturated by an inoculation dose of 5 x 10^4^ IFUs. The saturation can be caused by either the limited number of cells available for *C*. *muridarum* infection or an active suppression of *C*. *muridarum* replication in the mouse GI tract. Nevertheless, mice inoculated with either 5 x 10^4^ or 1 x 10^7^ IFUs developed similar but significant levels of live organism shedding in the rectal swabs throughout the experiments. These continuous live organism sheddings from the GI tracts did not cause any significant infection in the genital tracts, suggesting that under the experimental condition, the GI tract chlamydial organisms even at their maximal shedding levels were not able to autoinoculate the genital tracts.

The conclusion on the lack of autoinoculation by the GI tract *C*. *muridarum* into the genital tract was only based on the *C*. *muridarum* murine model. We should be cautious in applying the knowledge learnt from mice to *C*. *trachomatis* infection in humans. This is because not only the biology of both the organisms and hosts is different, but also the human behaviors differ significantly from those of mice. For example, if a person use fingers to touch both the anorectum and vagina, the chance of chlamydial spreading from the GI to genital tract is high. To evaluate whether this route of chlamydial spreading can enhance chlamydial pathogenicity in the genital tract, sexual behaviors-related clinical studies should be carried out.

It is worth noting that the lack of effective autoinoculation of *C*. *muridarum* from the GI into the genital tracts as demonstrated above does not necessarily mean that the GI tract *Chlamydia* cannot contribute to the chlamydial pathogenicity in the upper genital tract. On the contrary, what we have learnt from the current study may guide us to the correct direction for further investigating chlamydial pathogenesis. We hypothesize that besides the direct autoinoculation, indirect mechanisms may play significant roles. For example, the long-lasting pathology in the upper genital tract is accompanied with chlamydia-specific host responses [23, 24, 37, 38]. It will be interesting to investigate whether and how the host responses are induced and gut microbiota altered by the chlamydial infection in the GI tract may promote the chlamydial pathogenicity in the upper genital tract.
